# Microincision vitrectomy surgery: experimental visualization and quantification of vitreous contamination

**DOI:** 10.1186/s12886-020-01712-6

**Published:** 2020-11-10

**Authors:** Yumiko Machida, Hiroyuki Nakashizuka, Jun Shoji, Hiroyuki Shimada

**Affiliations:** Division of Ophthalmology, Department of Visual Sciences, Nihon University School of Medicine, Nihon University Hospital, 1-6 Kandasurugadai, Chiyoda-ku, Tokyo 101-8309 Japan

**Keywords:** Microincision vitrectomy surgery, Vitreous contamination, Vitrectomy, Postoperative endophthalmitis, Contamination, Fluoresbrite carboxylate microspheres, Fluorophotometry, Intraocular fiber catheter

## Abstract

**Background:**

To visualize and quantify vitreous contamination following microincision vitrectomy surgery (MIVS) using an experimental vitreous contamination model (EVCM).

**Methods:**

Enucleated porcine eyes with fluoresbrite carboxylate microspheres applied to the conjunctival surface were used as a type 1 EVCM. Twenty-five- or 27-gauge (G) trocar cannulas were inserted through the conjunctiva and sclera, followed by the placing and opening of an infusion cannula. These procedures were monitored by an intraocular fiber catheter. Secondly, condensed microspheres were applied to an excised sheet of porcine sclera to serve as type 2 EVCM. Twenty-five- or 27-G trocar cannulas were inserted perpendicularly through the top of the sclera where the condensed microspheres were applied, an infusion cannula was inserted, 0.1 mL of saline solution injected through the infusion cannula, and samples collected. The fluorescence strength of samples was then measured using fluorophotometry.

**Results:**

We visually detected fluorescent microspheres in 10/10 eyes with 25-G and 10/10 with 27-G MIVS. In the experimental quantification study, each MIVS gauge value was significantly higher than the control (*P* < 0.01). However, there was no significant difference between 25-G and 27-G MIVS.

**Conclusions:**

MIVS carries the risk of introducing contamination directly into the eyes when the trocar cannula is inserted and infusion cannula is opened, even when a 27-G MIVS is used. Our study has shown it is essential that the surgeon be aware of the possibility of introducing contamination from the conjunctiva at all times during MIVS.

**Supplementary Information:**

The online version contains supplementary material available at 10.1186/s12886-020-01712-6.

## Background

Recently, microincision vitrectomy surgery (MIVS) has become more common, making vitrectomy a safer procedure. However, serious complications, such as postoperative retinal detachment and postoperative endophthalmitis, are still sporadically observed. Postoperative endophthalmitis is the most serious complication after vitrectomy, and likely to cause serious vision loss.

When 25-G MIVS was first developed and its use broadened, it was reported that endophthalmitis occurred more frequently with MIVS than with conventional 20-G vitrectomy. Endophthalmitis was reported to be 12 or 28 times more likely to develop after 25-G MIVS than 20-G MIVS [1, 2]. More recently, the incidence of post–MIVS endophthalmitis seems to have reduced due to preventative strategies, however, even in the latest review of endophthalmitis after 25-G MIVS, the occurrence rate of endophthalmitis has still been higher than that of 20-G vitrectomy. In 2010, Oshima et al. developed 27-G vitrectomy [3], and it has been gaining popularity, however, the postoperative endophthalmitis rate has not yet been reported.

Tominaga et al. [4] reported the bacterial detection rate from the vitreous immediately after sclerotomy was 22.5% in the 25-G vitrectomy group with a two-step incision procedure, which was significantly higher than the 20-G group (2.4%). This observation suggested conjunctiva-residing bacterium may be introduced into the eye in MIVS during the sclerotomy, but imaging data is lacking. We think it is important for the vitreous surgeon to have the clear images to understand how bacterial contamination can be introduced into the eye during MIVS.

In this study, we clearly visualized the mechanism of contamination and quantified the contamination of 25-G and 27-G MIVS using our previously described experimental model [5].

## Methods

**Fig. 1:**
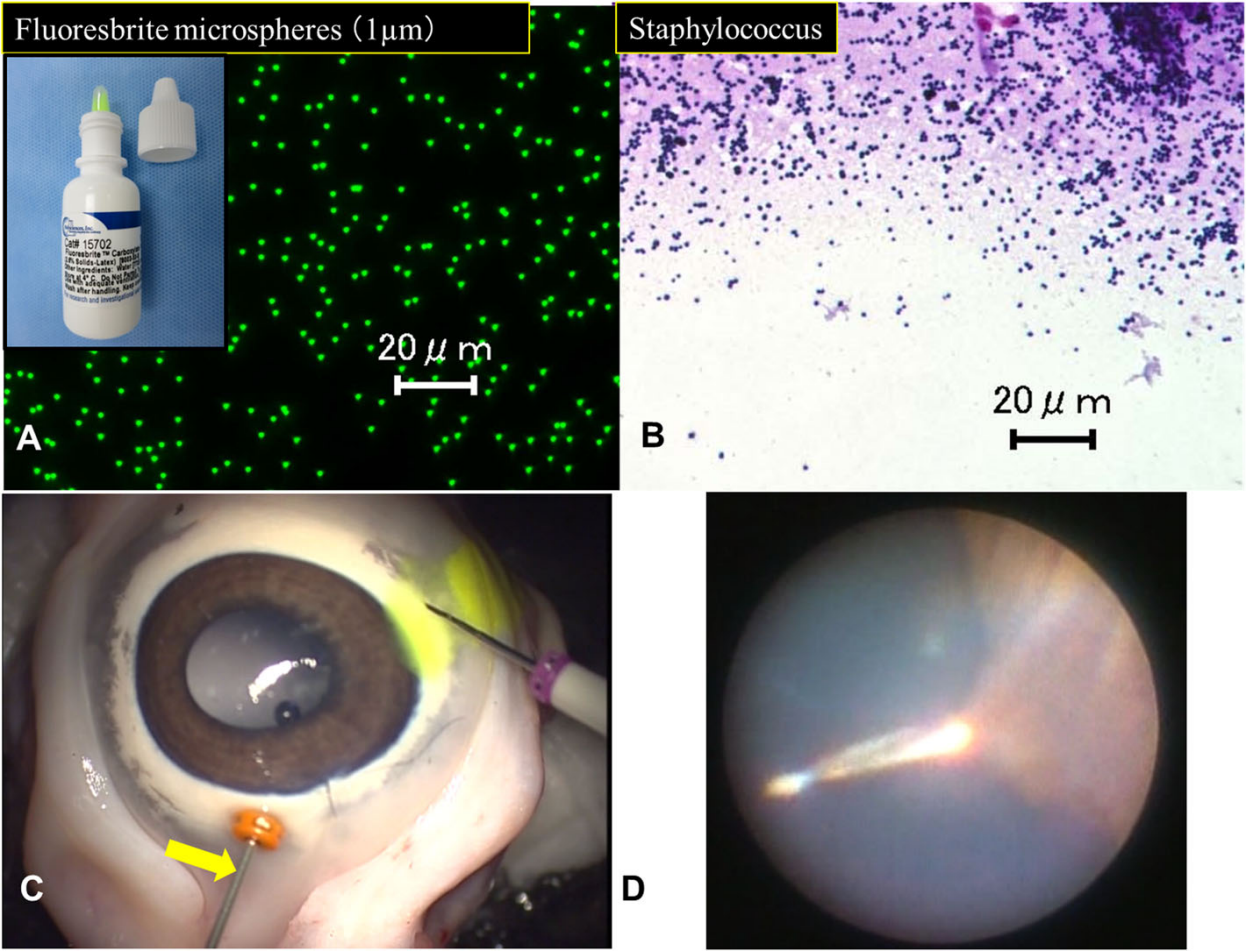
Enucleated porcine eyes served as a type 1 EVCM. **a**, **b** Fluoresbrite Carboxylate Microspheres (1.0 μm) are almost the same size as *Staphylococcus* bacteria. **c**, **d** Fluoresbrite carboxylate microspheres (2.5% Solids-Latex; Polysciences, Inc.) were applied to the conjunctival surface at the insertion site. Twenty-five- or 27-gauge (G) trocar cannulas (27-G in this image) were inserted through the conjunctiva and sclera, followed by the placing and opening of the infusion cannula. These procedures were carefully monitored with an intraocular fiber catheter (yellow arrow). Scale bars, 20 μm

Freshly harvested eyes from pigs butchered at age 6 months were used. We initially used enucleated porcine eyes as a Type 1 experimental vitreous contamination model (EVCM). The porcine eyes were fixed onto a Styrofoam head model with pins. A 23-gauge (G) lighted ocular endoscope (AS-611; FiberTech Co., Ltd., Sakura-shi, Japan) was inserted through the pars plana at the 12-O’Clock position. After that, two or three drops of fluoresbrite carboxylate microspheres (2.5% Solids-Latex, Polysciences, Inc., Warrington, PA) were applied to the conjunctival surface at the insertion site. The diameter of Carboxylate Microspheres (1.0 μm) is similar to the size of *Staphylococcus* bacteria (Fig. 1a-b). Twenty-five- or 27-gauge (G) trocar cannulas were inserted through the conjunctiva and sclera perpendicularly, followed by the insertion and opening of an infusion cannula. Procedures were monitored with an intraocular fiber catheter and repeated 10 times (Supplementary video).

**Fig. 2:**
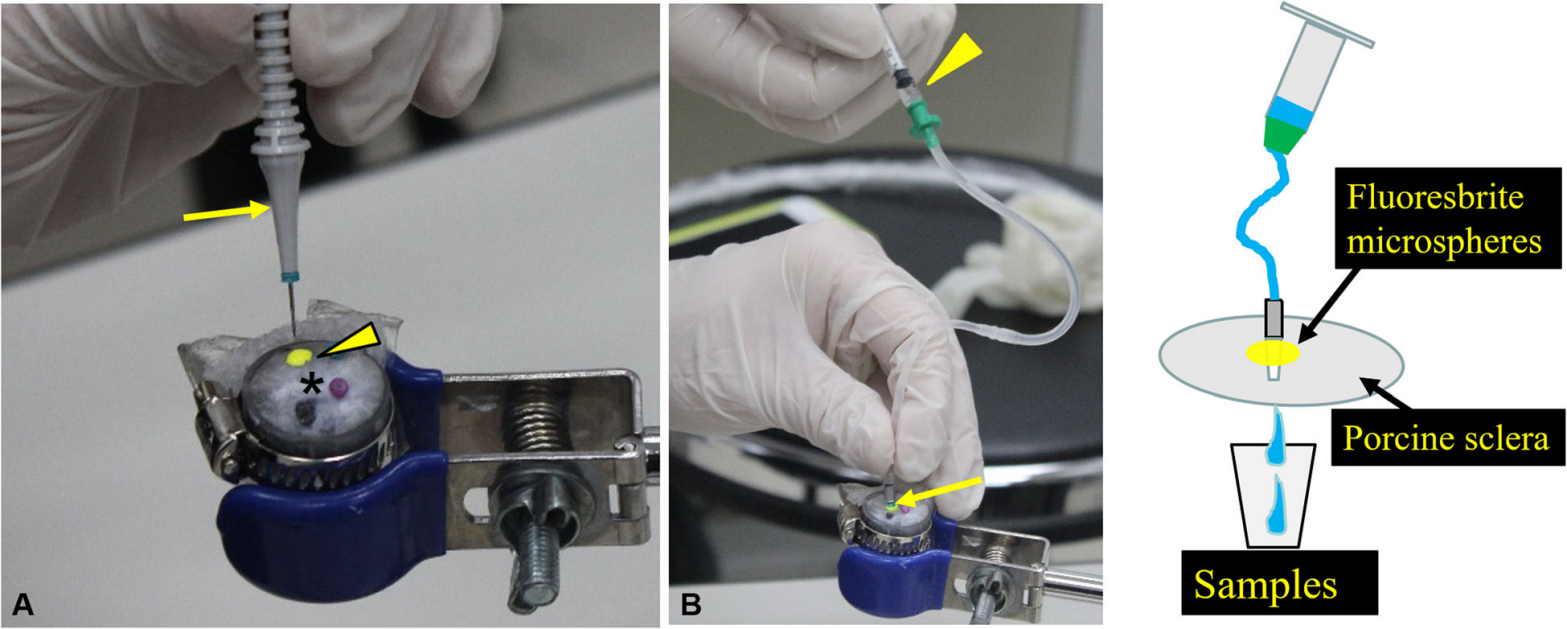
An excised sheet of porcine sclera as a type 2 EVCM. **a** For experimental quantification, 7 μl of condensed microspheres (arrowhead) were applied to an excised sheet of porcine sclera (asterisk). Twenty-five- or 27-G trocar cannulas (arrow) were inserted from the top of an applied condensed microsphere through the sclera. **b** An infusion cannula was filled with saline solution (arrow), then 0.1 mL of saline solution (arrowhead) was injected through infusion cannula, and samples collected

For experimental quantification, a Type 2 experimental vitreous contamination model was developed: Condensed microspheres were prepared by centrifugation for 20 min at approximately 5000 rpm, and 7 μL of the condensed microspheres were applied to an excised sheet of porcine sclera. Twenty-five- or 27-G trocar cannulas were then inserted perpendicularly through the top of the sclera where the condensed microspheres were applied. An infusion cannula filled with saline solution was then connected, and 0.1 mL of saline solution injected through the infusion cannula, and samples collected. As a control, 0.1 mL of saline solution was injected through sclera with a 27-G cannula without applying the microspheres. These procedures were repeated 10 times each for samples and controls. The fluorescence strength of the samples and controls were measured using fluorophotometry (Multi Label Counter ARVOmx 1420; PerkinElmer, Inc., Waltham, MA; Fig. 2b), with each specimen measured twice. Excitation and emission wavelengths were 485 nm and 535 nm, respectively. The average value for each sample were used for statistical analyses. The Steel-Dwass test for multiple comparisons was used for statistical analyses of three samples (25-G sample, 27-G sample and 27-G control). Statistical analyses were performed using MAC Toukeikaiseki Ver. 2 (ESUMI Co Ltd., Tokyo, Japan).

## Results

**Fig. 3:**
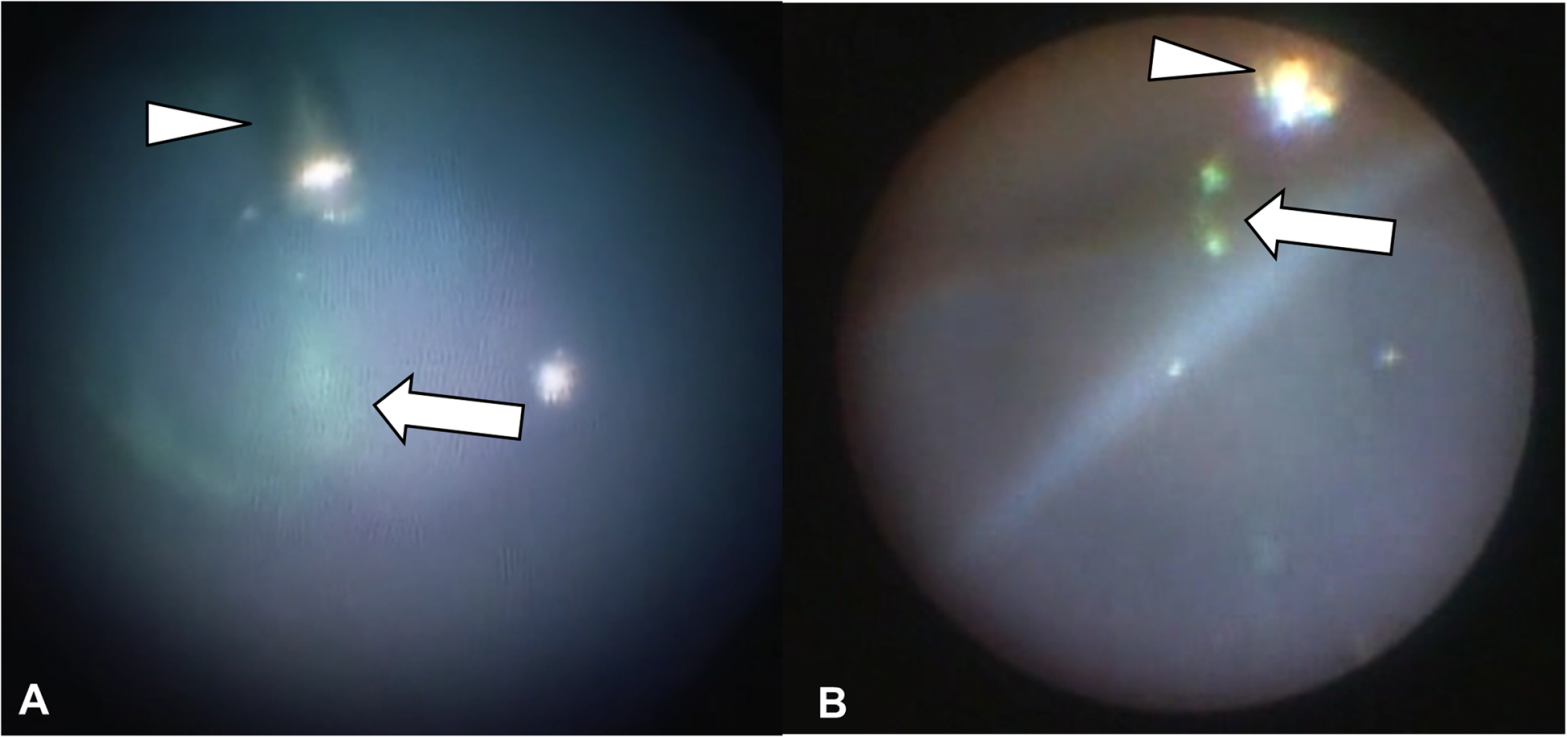
These procedures were carefully monitored using an intraocular fiber catheter (**a**, **b**). With the injection of saline solution, the microsphere particles were injected through the infusion cannula (arrowhead) and then spread into the vitreous cavity (arrow). We visually detected fluorescent microspheres in 10/10 eyes injected through a 25-G-, and 10/10 with a 27-G, cannula

**Fig. 4:**
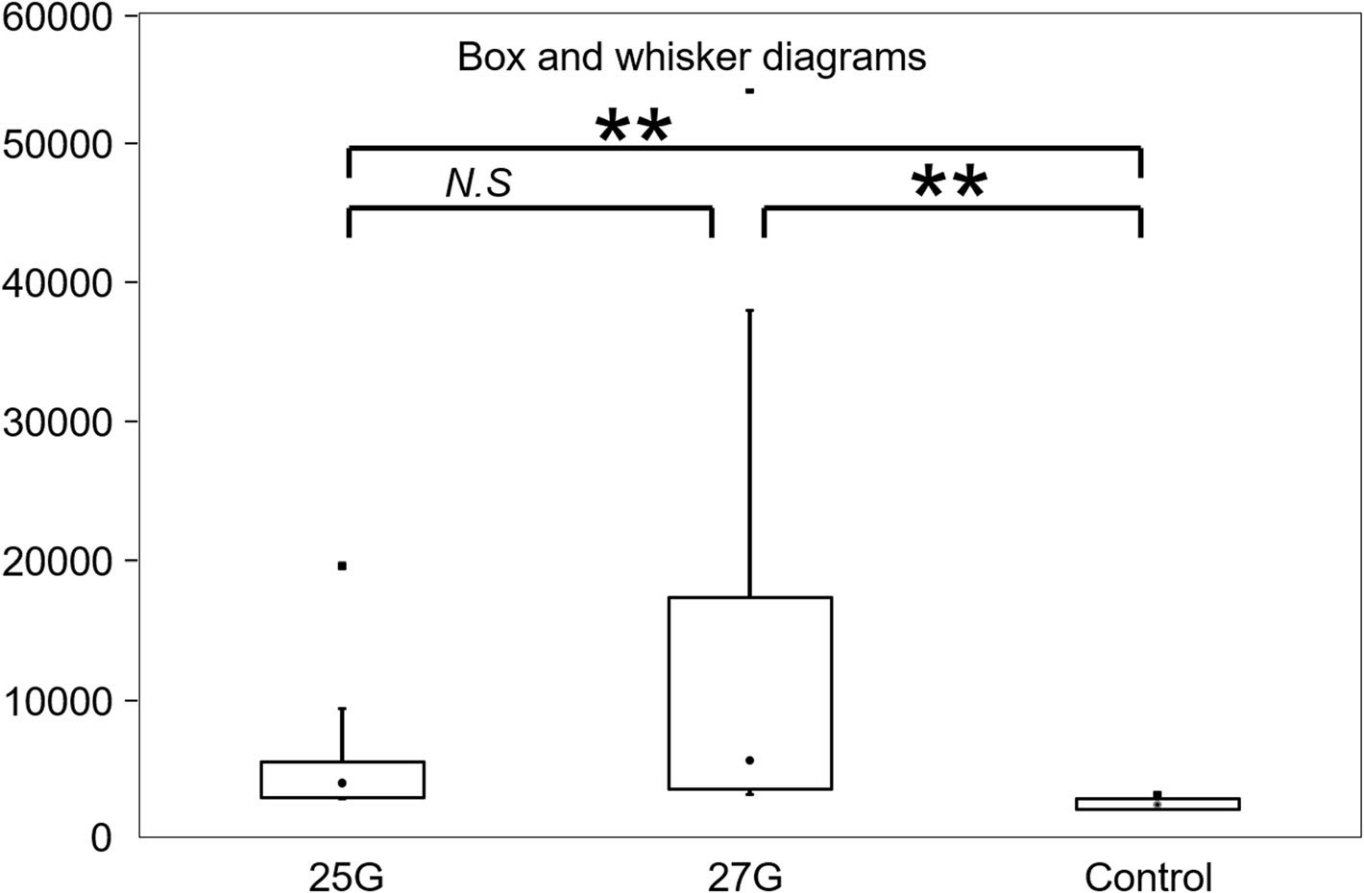
The Steel-Dwass test for multiple comparisons was used for statistical analyses of three samples. **, *P* < 0.01; N.S, Not significant

We visually detected fluorescent microspheres in all eyes injected through the 25-G and 27-G cannulas (10/10 for both the 25-G and 27-G; Fig. 3). When the cannula was opened, the microspheres spread into the vitreous and remained around the infusion site. In the experimental quantification study, the average fluorescence strength across samples and control were: 5.51 ± 4.87 × 10^3^ for 25-G; 12.60 ± 14.98 × 10^3^ for 27-G; and 2.45 ± 0.39 × 10^3^ for control (mean ± standard deviation), (Supplementary file 1). The samples collected with each trocar gauge were significantly different from the control (*P* < 0.01, 25-G; *P* < 0.01, 27-G). However, there was no significant difference between 25-G and 27-G in regard to fluorescence strength (Fig. 4), (Supplementary file 2).

## Discussion

Positive bacterial culture from the conjunctiva have been detected in 13–28% of cataract surgery, even after disinfection with povidone-iodine [6], suggesting it is difficult to completely eliminate bacteria on the conjunctiva. Postoperative endophthalmitis after MIVS has two opportunities to develop; during and after the operation. Tominaga et al. found significantly higher bacterial cultures in vitreous collected soon after trocar insertion with a 25-G compared with a 20-G [4], suggesting conjunctival bacteria are introduced during trocar insertion. It is thought that indigenous bacteria on the conjunctiva penetrate into the eye causing the endophthalmitis, as the pathogenic bacteria of endophthalmitis has been reported to be identical to normal conjunctival bacteria flora [7].

In the present study, microsphere penetration into the eye was visually confirmed in all cases using a 25-G and 27-G cannula. With opening of the irrigation canula, spreading of microspheres around the cannula was especially evident. Furthermore, when microspheres were applied on the conjunctiva in a quantitative study, significantly higher fluorescence intensities were obtained from samples compared to controls. These indicate that contamination on the conjunctiva adheres to the trocar and cannula with trocar insertion, and that the open irrigation cannula enables dispersal into the eye.

While not all contamination introduced into the eye is bacterial, previous studies have shown endophthalmitis can develop from just a few bacteria in the vitreous body [8]. Thus, the risk of postoperative endophthalmitis might be increased with MIVS as it inserts a trocar through the conjunctiva. While the irrigation volume could be sufficiently achieved using 20-G, the total irrigation volume will decrease due to the small size of cannula in MIVS. It has been reported that the lower flow volume through the 25-G infusion during surgery as compared with the 20-G cannula, at an approximately 7-fold lower infusion rate, may diminish the washing effect of the infusion [9]. Furthermore, if the peripheral vitrectomy was not performed effectively to allow wound self-closure, it might result in a further reduction in irrigation volume. A report by Tominaga et al. found complete peripheral vitrectomy resulted in a 0% positive bacterial culture rate in both 20-G and 25-G groups at the end of the operation [4]. In other words, it is possible to remove bacteria adhering to the periphery of the cannula by performing peripheral vitrectomy. Our experiments also show that the contamination from the conjunctiva possibly remains, particularly near the cannula, and that peripheral vitrectomy near the cannula is important in preventing endophthalmitis.

Shimada et al. proposed the concept of intraoperative disinfection that exploited the advantages of shorter disinfection times with 0.25% PI and fewer side effects on the ocular surfaces [10]. With routine MIVS, they observed a bacterial detection rate of 2% from the vitreous cavity at the end of vitrectomy, but continuous ocular surface irrigation with 0.25% PI resulted in a bacterial detection rate of 0% at the completion of vitrectomy, without any occurrence of endophthalmitis. In MIVS, it is inevitable that contamination on the conjunctiva will penetrate into the eye when the trocar is inserted. However, we are able to prevent the introduction of live bacteria. Therefore, it is of great importance that the ocular surface is disinfected just before insertion of trocar, such as by applying disinfecting agents like 0.25%PI. It is possible that intraoperative infection can be reduced by such intraoperative infection countermeasures. Additionally, postoperative infection can also be reduced by performing sclerotomies by oblique insertion [11] and using an air tamponade to close the inner valve securely [12], thus lowering the chance of postoperative infection. These measures have lowered the current rate of postoperative endophthalmitis with MIVS [13]. Our study is the first to visually demonstrate that contamination can be brought into vitreous cavity through conjunctiva, even with 27-G MIVS. It is essential that the surgeon be aware of the possibility of introducing contamination on the conjunctiva at all times during surgery, especially when they insert the trocar at the beginning of the MIVS.

The present study has limitations. First, porcine eyes were used as an alternative model for human eyes. The porcine eye has a thicker sclera than the human eye. However, a histological study revealed human and porcine scleras to have a similar histology and collagen bundle organization, indicating that the porcine sclera can serve as a good model for the human sclera [14]. In the human eye, which has a thinner sclera, significantly more bacteria might be introduced, such that our model might underestimate the severity of contamination. Second, the number of experimental cases was small. Yet, we visually detected fluorescent microspheres in 100% of eyes, and in the experimental quantification study, the fluorescence strength averages differed significantly between the samples and controls. Therefore, we consider the sample size of this study to have been sufficient. Third, in the experimental quantification study, only 0.1 mL saline solution was injected through infusion canula to obtain samples containing fluoresbrite carboxylate microspheres, and this volume might have been insufficient to wash out all the microspheres adhered to the inner wall of cannula. This might be a reason there was no statistically significant difference between 25-G and 27-G.

However, we succeeded in demonstrating that trocar insertion carries a risk of introducing contamination directly into the eyes mechanically, even when a 27-G trocar is used. Thus, disinfection of surgical fields with povidone iodine during surgery seems to be the best way to prevent endophthalmitis after MIVS. The supplementary video will be beneficial for the vitreous surgeon, providing the clear images of how bacteria are introduced into the vitreous cavity during MIVS. Our study has shown it is essential that the surgeon be aware of the possibility of introducing contamination from the conjunctiva at all times during MIVS.

## Supplementary Information

**Additional file 1 **Supplemental Digital Content 1: **Video S1.** Enucleated porcine eyes served as a type 1 EVCM. First, an intraocular fiber catheter was inserted at the 12 O’Clock position. Fluoresbrite Carboxylate Microspheres were applied to the conjunctival surface at the insertion site. Then, a 27-gauge trocar cannula was inserted through the conjunctiva and sclera. An infusion cannula was connected to the trocar cannula. With the opening of infusion cannula, the microsphere particles spread into the vitreous cavity.

**Additional file 2.**

**Additional file 3.**

## Data Availability

The datasets used and analyzed during the current study are available from the corresponding author on reasonable request.

## Abbreviations

MIVS: microincision vitrectomy surgery
EVCM: experimental vitreous contamination model
