# Supplementary material for: Microincision vitrectomy surgery: experimental visualization and quantification of vitreous contamination
Source: BMC Ophthalmol. 2020 Nov 10;20:441. doi: 10.1186/s12886-020-01712-6 (PMC7653715; doi:10.1186/s12886-020-01712-6)
Supplement: Supplementary file 2 — Additional file 2. [file 12886_2020_1712_MOESM2_ESM.docx]

| **The original data** | | | |
| --- | --- | --- | --- |
| No | 25G | 27G | Control（27G） |
| 1 | 2765 | 5202 | 3011 |
| 2 | 4353 | 16706 | 3075 |
| 3 | 19720 | 19650 | 2698 |
| 4 | 3162 | 3168 | 2774 |
| 5 | 3574 | 5900 | 2025 |
| 6 | 5102 | 3124 | 2198 |
| 7 | 2826 | 3659 | 2077 |
| 8 | 4178 | 4406 | 1988 |
| 9 | 6517 | 9813 | 2499 |
| 10 | 2891 | 54357 | 2219 |

| **Fundamental statistic** | | | |
| --- | --- | --- | --- |
|  | 25G | 27G | Control (27G) |
| Number | 10 | 10 | 10 |
| Total | 55089.6667 | 125986.667 | 24565.6667 |
| Average | 5508.96667 | 12598.6667 | 2456.56667 |
| Harmonic mean | 3957.90943 | 5784.59466 | 2396.79493 |
| Geometric mean | 4450.06841 | 7822.2968 | 2426.18293 |
| Median | 3876.16667 | 5551.16667 | 2359.16667 |
| 25th percentile | 2874.91667 | 3536.41667 | 2064.16667 |
| 75th percentile | 5455.91667 | 17442.1667 | 2833.41667 |
| Maximum value | 19720.1667 | 54357.1667 | 3075.16667 |
| Minimum value | 2765.16667 | 3124.16667 | 1988.16667 |
| Sum of squared deviation | 237076714 | 2243436853 | 1523880.4 |
| Variance: n | 23707671.4 | 224343685 | 152388.04 |
| Variance: n-1 | 26341857.1 | 249270761 | 169320.044 |
| Standard deviation: n | 4869.05241 | 14978.1069 | 390.369107 |
| Standard devitation: n-1 | 5132.43189 | 15788.3109 | 411.485169 |
| Coefficient of variation: n | 0.88384133 | 1.18886445 | 0.15890841 |
| Coefficient of variation: n-1 | 0.93165056 | 1.25317316 | 0.16750417 |
| Range | 16955 | 51233 | 1087 |
