# Supplementary material for: Microincision vitrectomy surgery: experimental visualization and quantification of vitreous contamination
Source: BMC Ophthalmol. 2020 Nov 10;20:441. doi: 10.1186/s12886-020-01712-6 (PMC7653715; doi:10.1186/s12886-020-01712-6)
Supplement: Supplementary file 3 — Additional file 3. [file 12886_2020_1712_MOESM3_ESM.docx]

| **The Steel-Dwass test for multiple comparisons** | | | | | |
| --- | --- | --- | --- | --- | --- |
|  | Rank sum | statistic | 5% | 1% |  |
| 25G : 27G | 83.0 | -1.6630 | 2.3437 | 2.9138 | N. S |
| 25G : Control (27G) | 148.0 | 3.2505 | 2.3437 | 2.9138 | P < 0.01 |
| 27G : Control (27G) | 155.0 | 3.7796 | 2.3437 | 2.9138 | P < 0.01 |
|  |  |  |  |  | N.S: no significant difference |
